# *In vitro*-Induced Human IL-10^+^ B Cells Do Not Show a Subset-Defining Marker Signature and Plastically Co-express IL-10 With Pro-Inflammatory Cytokines

**DOI:** 10.3389/fimmu.2018.01913

**Published:** 2018-09-05

**Authors:** Laura C. Lighaam, Peter-Paul A. Unger, David W. Vredevoogd, Dorit Verhoeven, Ellen Vermeulen, Annelies W. Turksma, Anja ten Brinke, Theo Rispens, S. Marieke van Ham

**Affiliations:** ^1^Department of Immunopathology, Sanquin Research, Amsterdam, Netherlands; ^2^Landsteiner Laboratory, Amsterdam UMC, University of Amsterdam, Amsterdam, Netherlands; ^3^Swammerdam Institute for Life Sciences, University of Amsterdam, Amsterdam, Netherlands

**Keywords:** immune regulation, Breg, IL-10, IL-6, t-SNE analysis, B cell plasticity, cytokine co-expression, B cell activation

## Abstract

Regulatory B cells (Breg) have been described as a specific immunological subsets in several mouse models. Identification of a human counterpart has remained troublesome, because unique plasma membrane markers or a defining transcription factor have not been identified. Consequently, human Bregs are still primarily defined by production of IL-10. In this study, we sought to elucidate if *in vitro-*induced human IL-10 producing B cells are a dedicated immunological subset. Using deep immune profiling by multicolor flow cytometry and t-SNE analysis, we show that the majority of cells induced to produce IL-10 co-express pro-inflammatory cytokines IL-6 and/or TNFα. No combination of markers can be identified to define human IL-10^+^TNFα^−^IL-6^−^ B cells and rather point to a general activated B cell phenotype. Strikingly, upon culture and restimulation, a large proportion of formerly IL-10 producing B cells lose IL-10 expression, showing that induced IL-10 production is not a stable trait. The combined features of an activated B cell phenotype, transient IL-10 expression and lack of subset-defining markers suggests that *in vitro*-induced IL-10 producing B cells are not a dedicated subset of regulatory B cells.

## Introduction

A large number of studies demonstrated the existence of a regulatory subset of B cells (Bregs) in mice (1, 2). Still, it is difficult to pinpoint a unique Breg phenotype, as the unspoken consensus that murine Bregs are CD1d^Hi^CD5^+^CD19^+^ overlap in surface markers of B-1a cells (CD11b^low^CD5^+^IgD^+^), marginal zone B cells (CD1d^Hi^CD23^−^IgM^Hi^) and T2 marginal zone precursor B cells (CD1d^Hi^CD23^+^IgM^+^) (3). Indeed, IL-10 production seems to be the only distinguishing feature of Bregs, explaining their alias of “B10” cells (4). Once activated, IL-10-producing B cells can inhibit pro-inflammatory immune responses. The mechanisms of action of this process are largely unknown. Some of these regulatory effects act through IL-10, others can be IL-10 independent [reviewed by (5)]. It was shown that IL-10-producing B cells inhibit CD4^+^ T cell proliferation, increase FoxP3 expression in these cells and reduce T_H_1 skewing. These effects were not only dependent on secreted IL-10, but also on CD80/86 costimulatory signals (6–8).

In human, specific B cells were also shown to exhibit regulatory functions (9–16). The human equivalent of murine IL-10-producing Bregs however, remains matter of intense debate, both in terms of its actual existence and its potential defining phenotype as a specific B cell subset. Initially, human IL-10-producing B cells were described as being CD19^+^CD24^Hi^CD38^Hi^. In one report the majority of the IL-10^+^ B cells were also described as being IgM^Hi^IgD^Hi^CD1d^Hi^CD5^Hi^CD10^+^CD20^+^CD27^−^ (11). Another report showed that different subsets of transitional IL-10^+^CD24^Hi^CD38^Hi^ B cells have distinct regulatory properties (17). Other studies describe entirely different phenotypes for IL-10^+^ B cells, such as CD19^+^CD25^+^CD71^+^CD73^−^CD274^−^, CD24^Hi^CD27^+^CD48^Hi^CD148^Hi^ and CD20^+^CD27^+^CD43^+^CD11b^+^ (1, 18–20). Because the reported human IL-10-producing B cells show resemblance to immature, transitional, marginal zone, activated and memory B cells and even plasma cells, it is conceptually difficult to attribute a single regulatory function to these cells or coin them a Breg subset with confidence (1, 18–24). The marginal overlap between murine Bregs and human IL-10-producing B cells makes translation from animal studies to potential human Breg function all the more difficult.

Another challenging aspect of investigating *in vivo* relevance of IL-10-producing B cells is that IL-10 production by most B cells may not be an innate trait of a specific subset. Spontaneous IL-10 production in mice and men is only rarely reported, making the existence of ‘natural Bregs' controversial (19, 25). To properly assess the capacity of B cells to produce IL-10, *ex vivo* stimulation is routinely performed. Non-cognate triggering of B cells seems particularly potent in inducing IL-10, for example via TLR4 and TLR9 or via CD40L (11, 25–32). Other IL-10-inducing stimulations, such as IL-21, autoantigens, vitamin D3 and human chorionic gonadotropin (hCG) have been reported, but these have not gained broad recognition (33–35). Besides this non-cognate triggering, IL-10 can also be induced by B cell receptor (BCR) triggering (30), although data concerning simultaneous stimulation of BCR and TLR9 show conflicting results. In one study, simultaneous BCR ligation augmented CpG-induced IL-10 production (29). The opposite was found in another study with BCR ligation reducing the efficacy of CpG in inducing IL-10 in B cells, making it unclear what the effects of combined stimulations are on IL-10 production by B cells (3). In all of these cases, it is unclear whether Bregs develop from a specific pre-Breg lineage, or whether many B cells can adopt a regulatory phenotype after receiving the appropriate signals (36). The production of IL-10 by subsets resembling different B cell subtypes supports the latter theory. Finally, it has been shown that IL-10^+^ B cells can also produce the pro-inflammatory cytokine TNFα (37). Co-expression of different cytokines suggests IL-10 can be produced by a range of B cells and is not a trait of a dedicated IL-10 producing regulatory B cell.

It is important to realize, although often underappreciated, that in lymph nodes IL-10 can have decidedly immunoactivatory effects, especially on B cell differentiation and humoral immune responses. IL-10 reduces B cell apoptosis in germinal centers (GCs) and induces plasma cell differentiation (23, 38–40), antibody production and promotes Ig isotype switching (13, 40). Thus, in contrast to the proposed predominant regulatory role of Bregs on immunity, autocrine secretion of IL-10 by B cells is important in supporting humoral immune responses. Therefore, IL-10 may on the one hand be secreted by B cells at specific stages of B cell activation and function to direct immunity against specific antigens toward humoral immunity, while simultaneously acting as immune regulator for other arms of the immune system. The label “Breg subset” for IL-10 producing B cells would in that case be unfortunate and may give rise to undesired conclusions about identification of these cells in settings of human health or disease.

A true IL-10^+^ “Breg subset” would be expected to express some subset-defining, unique markers, transcription factors or other co-expressed regulatory molecules.

We therefore investigated the potential of B cells to stably produce IL-10 after stimulation with different agents, and investigated if they exhibit a unique and stable phenotype.

## Materials and methods

### Isolation of human B cells

Buffycoats of healthy human donors were obtained from Sanquin Blood Supply upon informed consent and approval by local ethical committee (Sanquin Amsterdam) and in line with the Declaration of Helsinki. Peripheral blood mononucleated cells (PBMCs) were isolated from buffycoats using a Lymphoprep (Axis-Shield PoC AS) density gradient. CD19^+^ cells were separated using magnetic Dynabeads (Invitrogen) following manufacturer's instructions; resulting in >98% purity.

### Cell lines

3T3 mouse fibroblast cells expressing human CD40L (41) were maintained in IMDM medium supplemented with fetal calf serum (FCS; 10%; Bodinco), penicillin (100 U/mL, Invitrogen), streptomycin (100 μg/mL, Invitrogen), β-mercaptoethanol (50 μM, Sigma-Aldrich), and G418 (500 μg/mL; Life Technologies) at 37°C in an atmosphere with 5% carbon dioxide. The day before experiments were conducted, cells were trypsinized, washed with B cell medium (RPMI medium supplemented with FCS (5%, Bodinco), penicillin (100 U/mL, Invitrogen), streptomycin (100 μg/mL, Invitrogen), β-mercaptoethanol (50 μM, Sigma-Aldrich), L-glutamine (2mM, Invitrogen), human apo-transferrin (20 μg/mL, Sigma-Aldrich) depleted for IgG using protein A sepharose (GE Healthcare), irradiated with 30Gy and allowed to attach overnight to flat bottom 96-wells culture plates (Thermo Scientific).

### Culturing of B cells and IL-10 induction

B cells were maintained in B cell medium at 37°C in an atmosphere with 5% carbon dioxide at a concentration of 5 ^*^ 10^6^/mL, in 96-wells culture plates (Greiner Bio-One). To induce IL-10 production, a range of stimuli were used: CpG (CpG ODN 2006; 1.25 μM; Invivogen; sequence: 5′-tcgtcgttttgtcgttttgtcgtt-3′), R848 (1 μg/mL; Alexis Biochemical), 20 μg/ml poly:IC (Sigma Aldrich), α-human IgM (Sanquin) or α-IgG (Sanquin) coated 3 μm polystyrene beads (used in a proportion of 2 beads to 1 B cell; Spherotech), 3T3-CD40L transfectants (41) (used in a proportion of 1 3T3-CD40L cell to 50 B cells).

### Flow cytometry and detection of IL-10

**Figure 1:**
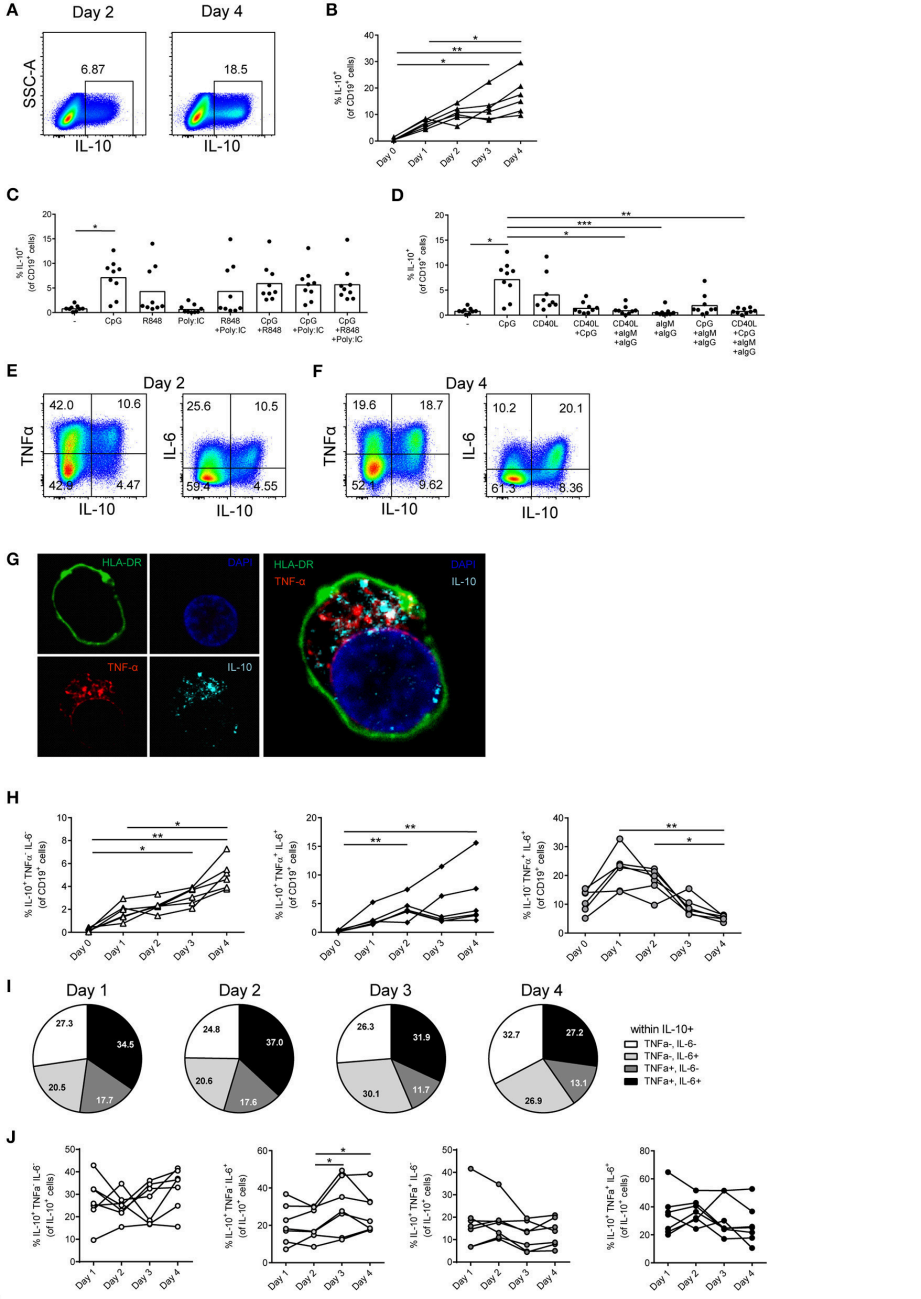
Most *in vitro*-induced human IL-10^+^ B cells also express TNFα and/or IL-6. **(A,B)** Total CD19^+^ B cells were isolated from blood and stimulated with CpG (1.25 μM) for 1–4 days followed by restimulation with PMA/Ionomycin/BFA (PIB) (*n* = 6). Representative dot plots are shown in **(A)**. **(C)** Induction of IL-10 by TLR stimuli. B cells were stimulated by 1.25 μM CpG, 1 μg/ml R848 and 20 μg/ml poly:IC to activate TLR9, TLR7/8, and TLR3, respectively (*n* = 9). **(D)** IL-10 induction in isolated CD19^+^ B cells stimulated for 4 days with 1.25 μM CpG, CD40L-expressing 3T3 cells (used in a ratio of 1 3T3-CD40L cell to 50 B cells), anti-IgM + anti-IgG beads (used in a ratio of 2 beads to 1 B cell) or left unstimulated and restimulated with PIB (*n* = 9). **(E,F)** Representative dot plots of IL-10, TNFα, and IL-6 producing B cells after 2 days **(E)** and 4 days of stimulation **(F)** with 1.25 μM CpG and PIB. **(G)** CLSM image of a CpG-stimulated B cell co-expressing TNFα and IL-10. Green: HLA-DR, blue: DAPI nuclear stain, red: TNFα, light blue: IL-10. Right panel: overlay. **(H)** Relative frequency at different time points of IL-10^+^TNFα^−^IL-6^−^, IL-10^−^TNFα^+^IL-6^+^, and IL-10^+^TNFα^+^IL-6^+^ cells after stimulation with 1.25 μM CpG and PIB (*n* = 6). Friedman test was performed. *p < 0.05. **p < 0.01. **(I,J)** Relative distribution of IL-10^+^TNFα^−^IL-6^−^ (white), IL-10^+^TNFα^−^IL-6^+^, IL-10^+^TNFα^+^IL-6^−^ (gray) and IL-10^+^TNFα^+^IL-6^+^ (black) B cells within IL-10^+^ B cells stimulated with 1.25 μM CpG at different time points (*n* = 7). **(I)** Averages of IL-10^+^ B cell subpopulations of seven donors are depicted as pie charts. **(J)** IL-10^+^ B cell subpopulations of individual donors are represented. Friedman test was performed. *p < 0.05. **(B–D,H,J)** Each dot/line represents 1 donor.

CpG-stimulated B cells were restimulated with PMA (100 ng/mL, Sigma-Aldrich), ionomycin (1 μg/mL, Sigma-Aldrich) and Brefeldin A (BFA; 10 μg/mL, Sigma-Aldrich) for 4 h on day of experiment. B cells were then washed with PBS and stained with LIVE/DEAD Fixable Near-IR (Dead cell stain kit, Invitrogen) for 30 min at room temperature in the dark. Subsequently, B cells were washed with PBS/1% bovine serum albumin (BSA; Sigma Aldrich). An extracellular staining was performed by incubating the B cells for 30 min at room temperature in the dark with fluorescently labeled antibodies (Supplemental Table 1). Cells were then fixed in 4% paraformaldehyde (PFA; Sigma-Aldrich), permeabilized with 0.5% saponin (Calbiochem) in PBS/1% BSA and stained with anti-IL-10, anti-TNFα and anti-IL-6 (Supplemental Table 1) antibodies for 30 min at room temperature in the dark. B cells were washed and resuspended in PBS/1% BSA and measured on an LSRII or LSRII Fortessa flow cytometer (BD Biosciences). Analysis was performed using FACS Diva (v6.1.2; BD Biosciences) and FlowJo (v7.6.5; Treestar) software. Live CD19^+^ cells were selected by setting a lymphocyte gate in FSC-A and SSC-A, followed by single cell gates using FSC-W/FSC-H and SSC-W/SSC-H and subsequent live CD19^+^ gating using the LIVE/DEAD Fixable Near-IR channel (APC-Cy7) and CD19 expression. Dead cells were excluded in Figures 1C,D in the absence of LIVE/DEAD Fixable Near-IR staining via strict gating on CD19^+^ cells. In all other figures, LIVE/DEAD Fixable Near-IR staining was used to exclude dead cells.

### Multimarker analysis using t-SNE

B cells were stimulated with CpG for 2 and 4 days, restimulated with PMA, ionomycin and BFA for 4 h on the day of experiment and stained with two different antibody mixtures (Supplemental Table 1). B cells were measured on LSRII Fortessa flow cytometer. Live B cells were gated in Flowjo v10.3 analysis software and exported as separate fcs-files for seven different donors. Populations were randomly down sampled to 13,000 events and subsequently concatenated into 91,000 events using plugins in Flowjo v10.3 to normalize contribution between donors. Next, concatenated samples were analyzed using the t-distributed stochastic neighborhood embedding (t-SNE) plugin in Flowjo v10.3. t-SNE is a non-linear dimension reduction technique creating a low-dimensional map of high-dimensional data that preserves distances between pairs of points (42). Basically, it “performs dimensionality reduction, allowing visualization of complex multi-dimensional data in fewer dimensions while still maintaining the structure of the data” (Flowjo v10 Documentation). This method has one main parameter, the perplexity value, which “sets the number of effective nearest neighbors” (Flowjo v10 Documentation). In our analysis, perplexity was set to 70, theta to 0.5, eta to 200 and iterations to 1,000. IL-10, TNFα, and IL-6 were selected as t-SNE parameters in Figures 3, 4 and Supplemental Figures 1A,B and the analysis was performed within live CD19^+^ cells. All markers including IL-10, TNFα, and IL-6 were selected as t-SNE parameters in Supplemental Figures 1C,D and the analysis was performed within IL-10^+^ B cells. Next, cytokine-positive cells (IL-10, TNFα, and IL-6) were manually gated in the concatenated sample file and used to distinguish the cytokine-positive subpopulations in the generated t-SNE data space. This allows to manually gate several IL-10^+^TNFα^−^IL-6^−^ populations and IL-10^+^ cell populations co-expressing TNFα and/or IL-6 in the t-SNE data space. Finally, expression of the cell surface markers, contained in the two antibody mixtures, on these cytokine-positive cell populations were visualized as half-offset overlay histograms. Quantification of cell surface marker expression was performed in the original non-down sampled, non-concatenated fcs data file of each individual donor.

### IL-10 secretion assay and cell sorting

Cells were cultured and restimulated with PMA and ionomycin. B cells were washed twice with cold (MACS) buffer containing PBS (Fresenius Kabi) with 0.5% Albuman (40 g/l) (Sanquin) and 2mM EDTA (Merck). B cells were then resuspended in 80 μl cold B cell medium per 10^7^ total cells and incubated with 20 μl IL-10 Catch Reagent (IL-10 secretion assay, Miltenyi Biotec) per 10^7^ total cells for 5 min on ice. Subsequently, warm (37°C) B cell medium was added at a concentration of 10^6^ cells/ml and cells were kept for 45 min at 37°C under slow continuous rotation. B cells were washed twice with cold (MACS) buffer, resuspended in 80 μl cold (MACS) buffer per 10^7^ total cells and incubated with 20 μl IL-10 Detection Antibody (PE) (IL-10 secretion assay, Miltenyi Biotec) per 10^7^ total cells for 10 min on ice. Next, B cells were washed with cold buffer and resuspended in PBS containing 1% BSA (Sigma-Aldrich). IL-10^+^ and IL-10^−^ B cells were analyzed on a LSRII flow cytometer (BD Biosciences) or isolated by FACS sorting on a FACS Aria (BD Biosciences).

### Confocal microscopy

Cells were cultured and restimulated with PMA, ionomycin and BFA for 4 h on day of experiment and stained with fluorescently labeled mAbs against HLA-DR-FITC (clone L243, BD Biosciences). Cells were then fixed in 4% PFA, permeabilized using 0.5% saponin and stained with 4′,6-diamidino-2-phenylindole (DAPI; Sigma-Aldrich), anti-TNF-DyLight549 (labeled in-house) and anti-IL-10-APC (clone JES3-19F1, BD Biosciences) antibody for 30 min in the dark. After staining, cells were left to attach to poly-L-Lysine-coated coverslips (BD Biosciences) for 90 min at 20°C. Coverslips were mounted on a microscope slide using VectaShield (Vector Labs), sealed with clear nail polish (Rimmel, London) and analyzed using a Leica TCS SP8 Confocal Laser Scanning Microscope (Leica). Acquired images were analyzed using Fiji software (build #3476, open source).

### Statistical analysis

Statistical analysis was performed using GraphPad Prism (version 6; GraphPad Software). Data were analyzed using a Wilcoxon test, or Friedman's test where appropriate. Results were considered significant at *p* < 0.05. Significance was depicted as ^*^(*p* < 0.05) or ^**^(*p* < 0.01).

## Results

### Most *in vitro*-induced human IL-10^+^ B cells also secrete TNFα and/or IL-6

Human CD19^+^ B cells isolated from peripheral blood were stimulated with the known IL-10 inducing TLR9 stimulus CpG (16, 29) and analyzed at different time points (Figures 1A,B). Within the first 4 days of stimulation the frequency of IL-10^+^ B cells steadily increased (Figure 1B). No further increase was observed at later time points (data not shown). Comparison of the efficacy of IL-10 induction by other reported antigen-independent TLR-stimuli (Figures 1C,D) demonstrated that IL-10 can indeed be induced by various stimuli, but that CpG stimulation was a potent and consistent *in vitro* inducer of IL-10 production by B cells in this culture system, both by itself or in combination with other TLR-stimuli (Figures 1C,D). In all conditions, the potency for IL-10 induction varied between donors. Antigen-dependent stimuli activating B cells via the B cell receptor (BCR) or CD40, showed similar IL-10 induction by CD40 and CpG, while BCR ligation did not significantly induce IL-10 (Figure 1D). Simultaneous addition of both CD40 and CpG did not yield a higher percentage of B cells expressing IL-10 (Figure 1D). To define the overall pro- or anti-inflammatory potential of the IL-10-producing B cells, co-expression of the cytokines TNFα and IL-6 was assessed (Figures 1E,F). A large proportion of IL-10 expressing B cells co-express TNFα and/or IL-6 (Figure 1E) that mostly does not reside in the same intracellular vesicles (Figure 1G). The percentage of IL-10^+^TNFα^−^IL-6^−^ single-positive B cells and IL-10^+^TNFα^+^IL-6^+^ triple-positive B cells increased over time (Figure 1H). In contrast, IL-10^−^TNFα^+^IL-6^+^ double-positive B cells were induced within 1 day of B cell stimulation, but diminished in frequency after that time point (Figure 1H). Co-expression of TNFα and/or IL-6 within the IL-10^+^ B cell population remained relatively stable over time (Figure 1I) without a significant decrease or increase in IL-10^+^TNFα^+^IL-6^−^ and IL-10^+^TNFα^−^IL-6^+^ double-positive populations (Figure 1J). These data show that most CpG-stimulated peripheral blood human B cells express IL-10 together with TNFα and/or IL-6.

### IL-10 and pro-inflammatory cytokine production by transitional B cells and plasmablasts

**Figure 2:**
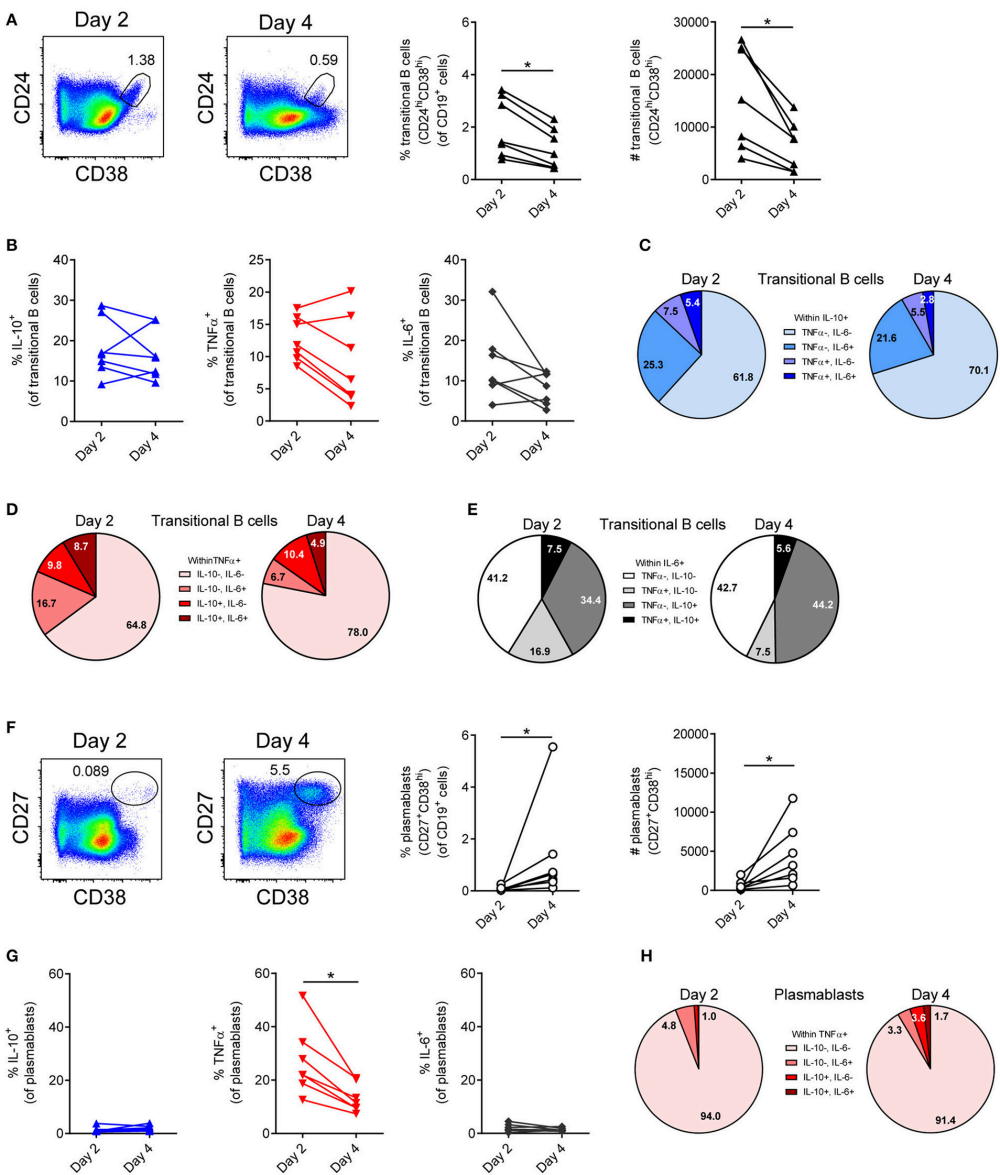
IL-10, TNFα, and IL-6 production by transitional B cells and plasmablasts after B cells were stimulated with CpG. **(A,F)** Frequency and number of transitional B cells **(A)** and plasmablasts **(F)** were quantified 2 and 4 days after B cells were stimulated with 1.25 μM CpG (*n* = 7). Representative dot plots are depicted. Each dot/line represents 1 donor. **(B,G)** Frequency of cytokine-producing cells within transitional B cell **(B)** and plasmablast populations **(G)** (*n* = 7). **(C–E)** Relative distribution of **(C)** IL-10^+^TNFα^−^IL-6^−^ (light blue), IL-10^+^TNFα^−^IL-6^+^ (blue), IL-10^+^TNFα^+^IL-6^−^ (lavender blue), IL-10^+^TNFα^+^IL-6^+^ (dark blue) B cells within IL-10^+^ transitional B cells; **(D)** IL-10^−^TNFα^+^IL-6^−^ (pink), IL-10^−^TNFα^+^IL-6^+^ (light red), IL-10^+^TNFα^+^IL-6^−^ (red) and IL-10^+^TNFα^+^IL-6^+^ (bordeaux) B cells within TNFα^+^ transitional B cells; **(E)** IL-10^−^TNFα^−^IL-6^+^ (white), IL-10^−^TNFα^+^IL-6^+^ (light gray), IL-10^+^TNFα^−^IL-6^+^ (gray) and IL-10^+^TNFα^+^IL-6^+^ (black) B cells within IL-6^+^ transitional B cells stimulated with 1.25 μM CpG at different time points (*n* = 7). **(H)** Relative distribution of IL-10^−^TNFα^+^IL-6^−^ (pink), IL-10^−^TNFα^+^IL-6^+^ (light red), IL-10^+^TNFα^+^IL-6^−^ (red) and IL-10^+^TNFα^+^IL-6^+^ (bordeaux) B cells within TNFα^+^ plasmablasts stimulated with 1.25 μM CpG (*n* = 7). Wilcoxon tests were performed. *p < 0.05.

As both transitional B cells and plasmablasts have been described to produce IL-10 (11, 15), these cellular subsets were analyzed within the total B cell population after 2 and 4 days of stimulation with CpG (Figure 2). Transitional B cells (CD24^+^CD38^+^; Figure 2A) are low in frequency and absolute numbers within the total B cell pool, decrease over time and produce IL-10, TNFα and IL-6 (Figure 2B). Within the IL-10^+^ transitional B cell population a small majority of cells are IL-10^+^TNFα^−^IL-6^−^, while other cells co-express IL-6 and/or TNFα (Figure 2C). These IL-10^+^TNFα^−^IL-6^−^ transitional B cells only constitute 0.14-0.22% of the CD19^+^ cells, whereas the total population of IL-10^+^TNFα^−^IL-6^−^ cells constitute 2-5% of the CD19^+^ cells. Similar to the dominance of IL-10^+^TNFα^−^IL-6^−^ cells within the total IL-10 producing transitional B cells, the majority of TNFα producing cells are IL-10^−^TNFα^+^IL-6^−^, while the IL-6 producing transitional B cells are either IL-10^−^TNFα^−^IL-6^+^ or co-express IL-10 (IL-10^+^TNFα^−^IL-6^+^; Figures 2D,E).

CpG stimulation of isolated B cells yield few, but over time increasing plasmablasts (CD27^+^CD38^Hi^; Figure 2F), which are IL-10^−^TNFα^+^IL-6^−^ for the large majority (Figures 2G,H). These data indicate that *in vitro* stimulation of B cells by CpG induces IL-10 to a certain extent in transitional B cells, but not in plasmablasts.

### Heterogeneity in cytokine production within IL-10^+^TNFα^−^IL-6^−^ and IL-10^+^TNFα^+^IL-6^+^ B cells

**Figure 3:**
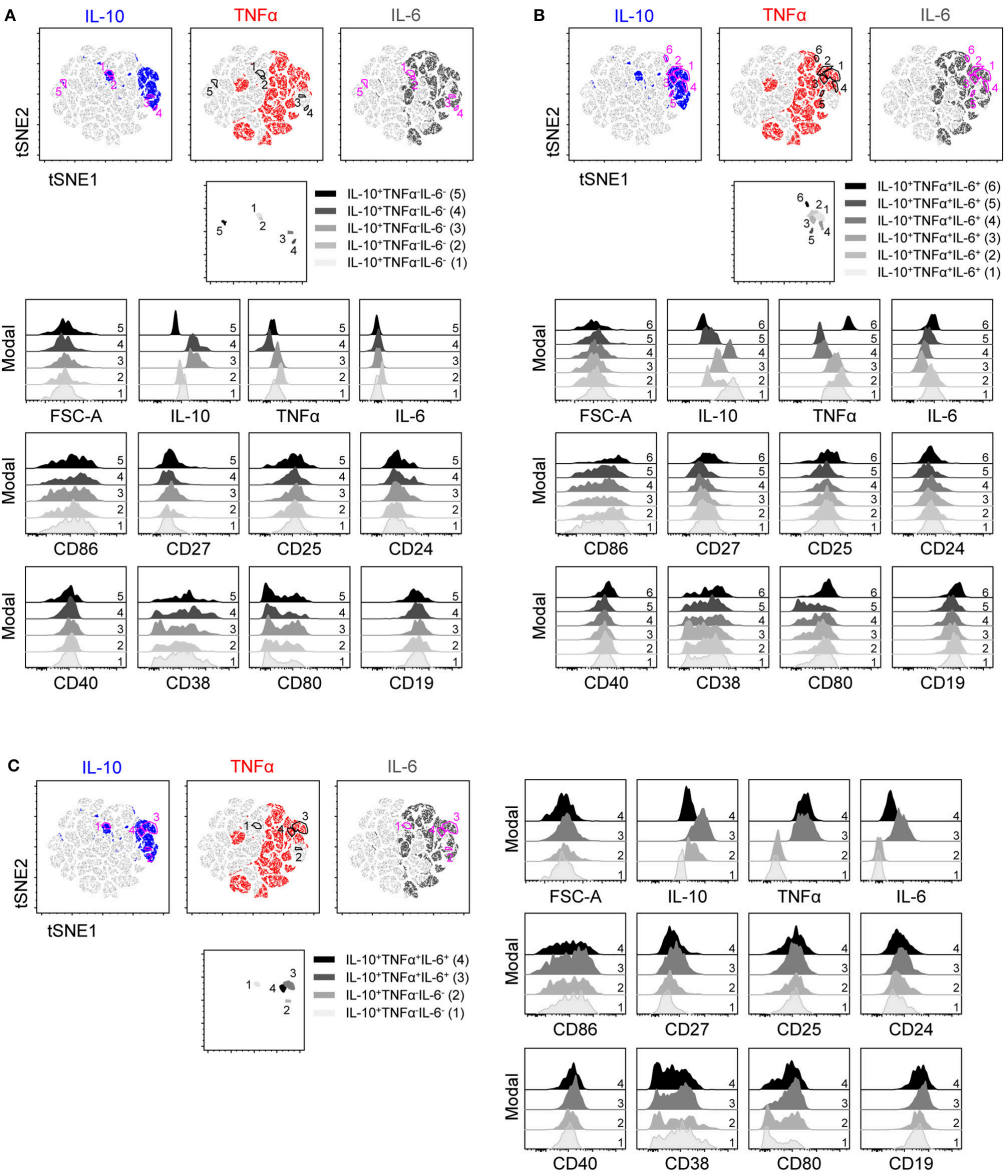
t-SNE clustering on IL-10, TNFα, and IL-6 within B cells stimulated with CpG for 2 days and the expression of co-stimulatory markers. t-SNE 2D scatter plot of 91,000 living single CD19^+^ B cells stimulated for 2 days with CpG and stained with staining mixture 1 containing antibodies against IL-10, TNFα, IL-6, CD86, CD27, CD25, CD24, CD40, CD38, CD80, and CD19 (*n* = 7). t-SNE settings were as followed: perplexity 70; theta 0.5; eta 200 and iterations 1,000. IL-10, TNFα, and IL-6 were selected as t-SNE parameters. Different colors depict the IL-10^+^ (blue), TNFα^+^ (red) and IL-6^+^ (dark gray) B cells. **(A–C)** Five IL-10^+^TNFα^−^IL-6^−^ **(A)**, six IL-10^+^TNFα^+^IL-6^+^ **(B)**, and two IL-10^+^TNFα^−^IL-6^−^ and IL-10^+^TNFα^+^IL-6^+^ **(C)** B cells were gated within the t-SNE 2D scatter plot, selected populations are depicted in a separate t-SNE 2D scatter plot and expression of various markers within these populations (represented as color spectrum of gray and black) were depicted in half-off set histograms.

**Figure 4:**
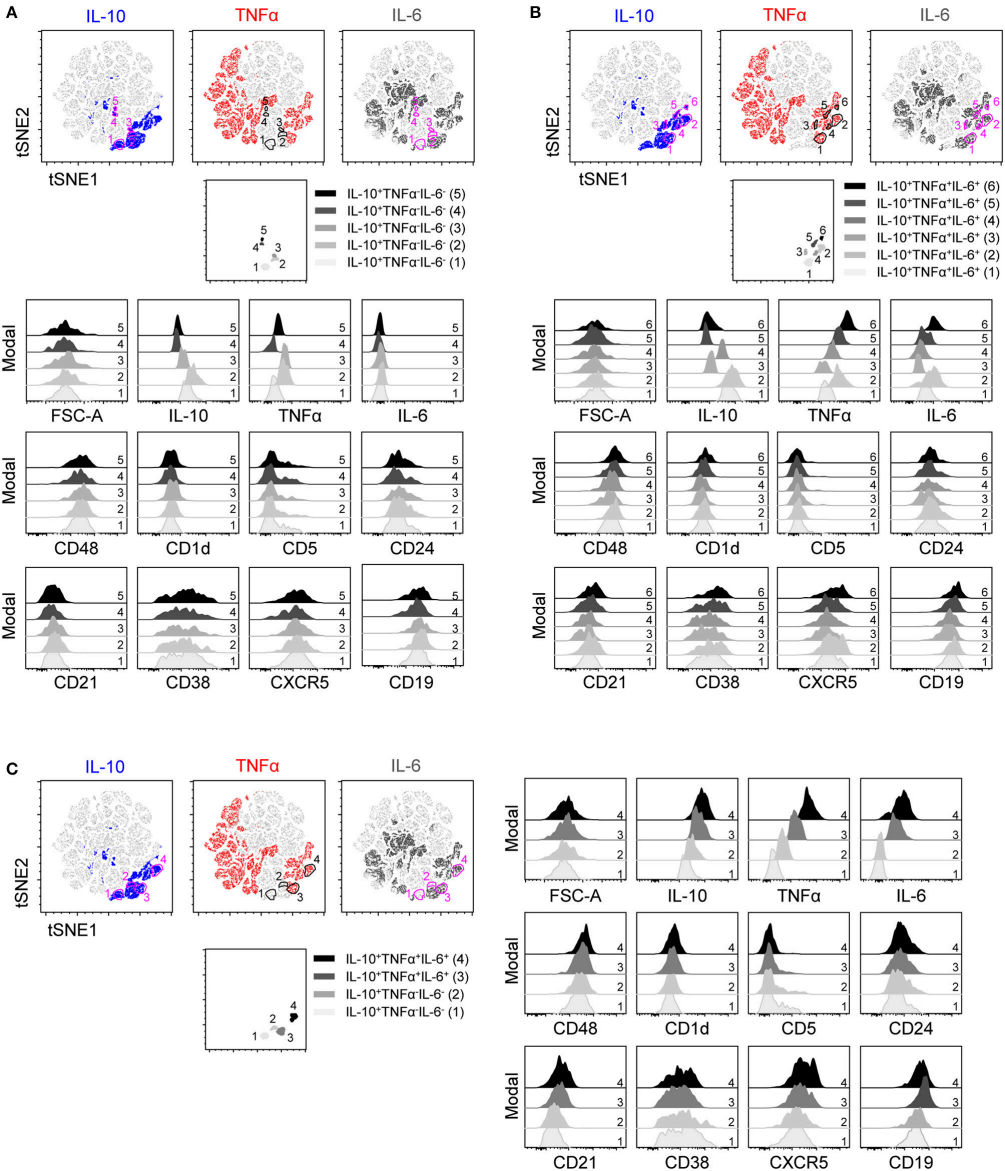
t-SNE clustering on IL-10, TNFα, and IL-6 within B cells stimulated with CpG for 2 days and the expression of B_reg_ associated markers. t-SNE 2D scatter plot of 91,000 living single CD19^+^ B cells stimulated for 2 days with CpG and stained with staining mixture 2 containing antibodies against IL-10, TNFα, IL-6, CD48, CD1d, CD5, CD24, CD21, CD38, CXCR5, and CD19 (*n* = 7). t-SNE settings were as followed: perplexity 70; theta 0.5; eta 200 and iterations 1,000. IL-10, TNFα, and IL-6 were selected as t-SNE parameters. Different colors depict the IL-10^+^ (blue), TNFα^+^ (red) and IL-6^+^ (dark gray) B cells. **(A–C)** Five IL-10^+^TNFα^−^IL-6^−^ **(A)**, six IL-10^+^TNFα^+^IL-6^+^ **(B)** and two IL-10^+^TNFα^−^IL-6^−^ and IL-10^+^TNFα^+^IL-6^+^ **(C)** B cells were gated within the t-SNE 2D scatter plot, selected populations are depicted in a separate t-SNE 2D scatter plot and expression of various markers within these populations (represented as color spectrum of gray and black) were depicted in half-off set histograms.

To identify if IL-10^+^TNFα^−^IL-6^−^ B cells and IL-10^+^ B cells co-expressing TNFα and/or IL-6 can be grouped into defined populations expressing unique markers, B cells were stimulated with CpG for 2 (Figures 3, 4) and 4 days (Supplemental Figures 1A,B), and analyzed for expression of markers previously associated with IL-10^+^ Bregs, either co-stimulatory markers (Figure 3; Supplemental Figure 1A) or other Breg associated markers (Figure 4; Supplemental Figure 1B) (1) using t-SNE. To separate all the cytokine^+^ subsets, IL-10, TNFα and IL-6 were used as parameters for the t-SNE clustering. Five different IL-10^+^TNFα^−^IL-6^−^ and six different IL-10^+^TNFα^+^IL-6^+^ B cell populations were identified showing varying expression levels of the cytokines analyzed (Figures 3, 4). IL-10 expression varied both within the IL-10^+^TNFα^−^IL-6^−^ (Figures 3A, 4A) and within the IL-10^+^TNFα^+^IL-6^+^ B cells (Figures 3B, 4B). In addition, some IL-10^+^TNFα^+^IL-6^+^ B cells express more IL-10 than IL-10^+^TNFα^−^IL-6^−^ B cells (Figures 3C, 4C; Supplemental Figures 1A,B). Together, these data indicate that IL-10 expression is not a defining trait of a specific B cell subset, but that the level of IL-10 expression varies in the different IL-10 producing B cells, independent of co-expression of TNFα and/or IL-6.

### CD21, CD80, and CD5 are differently expressed by, but not unique for, IL-10^+^TNFα^−^IL-6^−^ B cells

**Figure 5:**
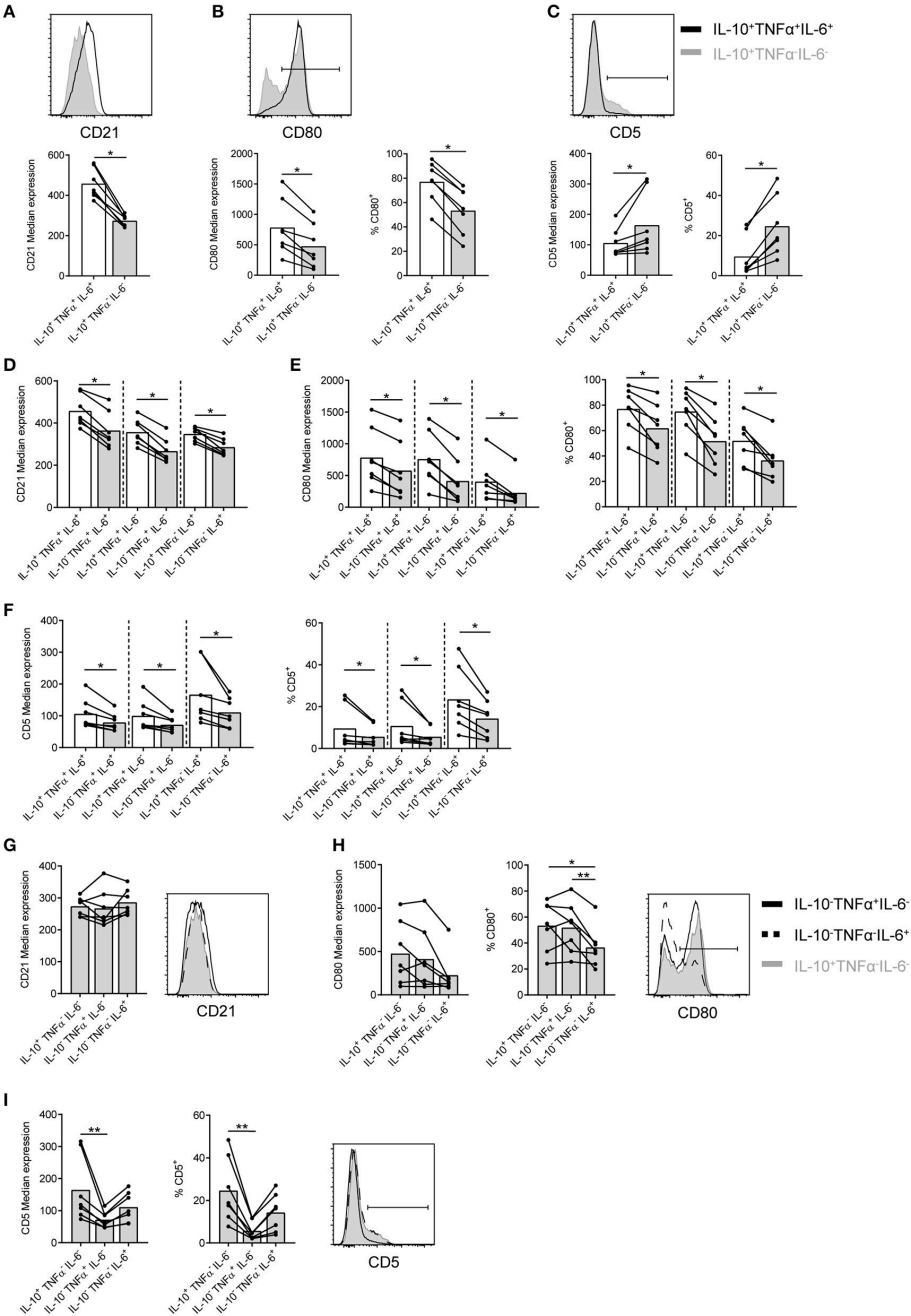
CD21, CD80, and CD5 are not unique markers for CpG-induced IL-10^+^TNFα^−^IL-6^−^ B cells. Total CD19^+^ B cells were isolated from blood and stimulated with CpG (1.25 μM) for 2 days followed by restimulation with PMA/Ionomycin/BFA (PIB) (*n* = 7). All live CD19^+^ cells were analyzed using flow cytometry. **(A–C)** Median expression of CD21, CD80, and CD5 on IL-10^+^TNFα^+^IL-6^+^ (white bar) and IL-10^+^TNFα^−^IL-6^−^ (gray bar) B cells. **(B,C)** Frequency of CD80^+^ and CD5^+^ B cells within IL-10^+^TNFα^+^IL-6^+^ and IL-10^+^TNFα^−^IL-6^−^ B cells. **(D–F)** Median expression of CD21, CD80, and CD5 on IL-10^+^TNFα^+^IL-6^+^ (white bar), various cytokine double-positive (IL-10^−^TNFα^+^IL-6^+^, IL-10^+^TNFα^+^IL-6^−^, IL-10^+^TNFα^−^IL-6^+^; as depicted) and IL-10^−^TNFα^+^IL-6^−^ (gray bar) and IL-10^−^TNFα^−^IL-6^+^ B cells (gray bar). **(E,F)** Frequency of CD80^+^ and CD5^+^ B cells within IL-10^+^TNFα^+^IL-6^+^ (white bar), various cytokine double-positive (IL-10^−^TNFα^+^IL-6^+^, IL-10^+^TNFα^+^IL-6^−^, IL-10^+^TNFα^−^IL-6^+^; as depicted) and IL-10^−^TNFα^+^IL-6^−^ (gray bar) and IL-10^−^TNFα^−^IL-6^+^ B cells (gray bar). Wilcoxon tests were performed. *p < 0.05. **(G–I)** Median expression of CD21, CD80 and CD5 on IL-10^+^TNFα^−^IL-6^−^ (gray bar), IL-10^−^TNFα^+^IL-6^−^ (gray bar) and IL-10^−^TNFα^−^IL-6^+^B cells (gray bar). (**H,I**) Frequency of CD80^+^ and CD5^+^ B cells within IL-10^+^TNFα^−^IL-6^−^ (gray bar), IL-10^−^TNFα^+^IL-6^−^ (gray bar) and IL-10^−^TNFα^−^IL-6^+^B cells (gray bar). Representative histograms are shown. Friedman test was performed. *p < 0.05. **p < 0.01. Each dot/line represents 1 donor.

Analysis of the markers previously associated with Bregs was performed to identify potential unique markers for IL-10^+^ B cells and to pinpoint if (and how) these markers discriminate for co-expression of IL-10 with TNFα and/or IL-6. No clear difference in marker expression between B cells stimulated with CpG for 2 and 4 days is observed (Figures 3, 4; Supplemental Figures 1A,B). Analysis of co-stimulatory markers or other cell surface markers coined as Breg markers in literature using t-SNE shows that IL-10^+^TNFα^−^IL-6^−^ B cells express lower levels of CD27, CD40, CD19, CD21, and CXCR5 than IL-10^+^TNFα^+^IL-6^+^ B cells (Figures 3C, 4C; Supplemental Figures 1A,B). In addition, the number of CD80^−^ B cells and CD5^+^ B cells are higher within the IL-10^+^TNFα^−^IL-6^−^ B population compared to the IL-10^+^TNFα^+^IL-6^+^ population (Figures 3C, 4C; Supplemental Figures 1A,B). After 2 days of stimulation with CpG (Figure 5), quantification of marker expression showed a trend for decreased expression of CD21, CD27, CD40, CD19, and CXCR5, but not the co-stimulatory marker CD86, on IL-10^+^TNFα^−^IL-6^−^ B cells compared to IL-10^+^TNFα^+^IL-6^+^ B cells (Figure 5A; Supplemental Figures 2A–E). In contrast, the frequencies of CD80^+^ and CD5^+^ B cells within the IL-10^+^TNFα^−^IL-6^−^ B cell population were clearly decreased and increased, respectively, compared to IL-10^+^TNFα^+^IL-6^+^ B cells (Figures 5B,C). Quantification of cytokine expression showed a trend that cytokine single-positive cells expressed lower levels of their respective cytokine compared to IL-10^+^TNFα^+^IL-6^+^ B cells (Supplemental Figures 2F–H, 4F–H). After 4 days of CpG stimulation only CD21 expression on IL-10^+^TNFα^−^IL-6^−^ B cells was significantly decreased compared to IL-10^+^TNFα^+^IL-6^+^ B cells (Supplemental Figure 3A). CD27, CD40, CD19, and CXCR5, but not CD86, showed a trend for decreased expression on IL-10^+^TNFα^−^IL-6^−^ B cells compared to IL-10^+^TNFα^+^IL-6^+^ B cells (Supplemental Figures 4A–E). Our data indicate that expression of multiple cytokines by B cells seems to correlate with an increased expression of activation markers. Therefore, we assessed whether co-expression of IL-10 in IL-10^−^TNFα^+^IL-6^−^ and IL-10^−^TNFα^−^IL-6^+^ single-positive or IL-10^−^TNFα^+^IL-6^+^ double-positive B cells correlates with increased expression of activation markers (Figures 5D–F; Supplemental Figures 2I–M, 3D–F, 4I–M). Indeed, co-expression of IL-10 showed an increased trend for the expression of CD21, CD27, CD40, CD19, CXCR5, and CD25 after 2 and 4 days of CpG stimulation (Figure 5D; Supplemental Figures 2I–M, 3D, 4I–M). In addition, the percentage of CD80^+^ cells increased with the co-expression of IL-10 (Figure 5E; Supplemental Figure 3E). Though, the frequency of CD5^+^ B cells did not increase with co-expression of IL-10 (Figure 5F; Supplemental Figure 3F). Taken together, this demonstrates that expression of two or more of the cytokines IL-10, TNFα and IL-6 may be an indication for increased B cell activation. Furthermore, although CD80, CD5 and CD21 show promise as markers to distinguish IL-10^+^TNFα^−^IL-6^−^ B cells from IL-10^+^TNFα^+^IL-6^+^ B cells, these are not unique markers for IL-10^+^TNFα^−^IL-6^−^ B cells as IL-10^−^TNFα^+^IL-6^−^ and IL-10^−^TNFα^−^IL-6^+^ B cells express similar levels of these markers (Figures 5G–I; Supplemental Figures 3G–I). In addition, the other activation and Breg associated markers are not unique for IL-10^+^TNFα^−^IL-6^−^ B cells (Supplemental Figures 2N–S, 4N–S). This is confirmed by t-SNE analysis within the IL-10^+^ B cells in which all surface markers together with IL-10, TNFα and IL-6 were used as parameters for t-SNE clustering (Supplemental Figures 1C,D). This demonstrates that IL-10^+^TNFα^−^IL-6^−^ B cells cluster relatively close with IL-10^+^ B cells co-expressing the pro-inflammatory cytokine IL-6, whereas IL-10^+^TNFα^+^IL-6^+^ B cells are relatively far from IL-10^+^TNFα^−^IL-6^−^ B cells. In addition, this t-SNE analysis shows that IL-10^+^TNFα^−^IL-6^−^ B cells do not express a unique set of surface markers. This was also observed when all surface markers excluding IL-10, TNFα, and IL-6 were used as parameters for t-SNE clustering (data not shown). Overall, the phenotype that distinguishes IL-10^+^TNFα^−^IL-6^−^ from IL-10^+^TNFα^+^IL-6^+^ are CD80^−^CD5^+^CD21^low^IL-10^low^CD27^low^CD40^low^CD19^low^CXCR5^low^, but does not define IL-10^+^TNFα^−^IL-6^−^ B cells.

### IL-10 production by *in vitro*-stimulated human B cells is a transient feature

**Figure 6:**
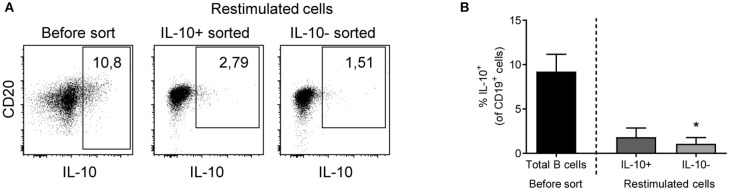
IL-10 expression by *in vitro-*stimulated human B cells is a transient trait. IL-10^+^ (dark gray) and IL-10^−^ (light gray) B cells were sorted on day 3 and re-cultured in B cell medium for 3 days. After PIB restimulation, IL-10 expression was assessed using intracellular staining (**B**; *n* = 3). Representative dot plots are shown **(A)**. Black bar depicts IL-10^+^ B cells before sort. Friedman test was performed. *p < 0.05.

To investigate if IL-10 production is a constitutive trait of a particular (regulatory) subset of B cells, or a transient feature of activated B cells, IL-10^+^ and IL-10^−^ B cells induced by CpG were sorted after 3 days, cultured for 3 additional days with B cell medium only and then re-stimulated. Most of the cells that were originally IL-10^+^ had lost their capacity to produce IL-10, while part of the formerly IL-10^−^ cells did produce IL-10 after this second round of stimulation (Figure 6A). Comparison of IL-10 production in the re-culture of IL-10^+^ and IL-10^−^ sorted cells showed similar percentages of cells expressing IL-10 (Figure 6B). This suggests that IL-10 production upon TLR9 stimulation represents a transient property of activated B cells.

## Discussion

To date, conflicting data on the phenotype and stability of human regulatory B cells lead to an ongoing debate on the functional regulatory importance of human Bregs. A few unique populations of human B cells have been identified that have all the characteristics of true regulatory cells, like CD24^Hi^CD27^+^, CD24^Hi^CD38^Hi^, and CD25^Hi^CD71^+^ B cells (36). This is in part confirmed by our data showing that CD24^Hi^CD38^Hi^ transitional B cells contain a large proportion of IL-10^+^TNFα^−^IL-6^−^ B cells. These IL-10^+^TNFα^−^IL-6^−^ transitional B cells only constitute 0.14–0.22% of the CD19^+^ cells, whereas the total population of IL-10^+^TNFα^−^IL-6^−^ cells constitute 2–5% of the CD19^+^ cells, indicating that the majority of IL-10^+^TNFα^−^IL-6^−^ cells consist of other B cell subsets. In general however, the main identifier of regulatory B cells is still considered to be IL-10 and in mice, indeed IL-10 seems to be expressed by B cell populations that are accepted to be Bregs (4). In human, B cells that produce IL-10 upon *in vitro* stimulation are therefore also often coined Bregs. Defined markers are however lacking and the stability of IL-10 production in these cells is unclear. There is continuous debate about the question if human IL-10-producing B cells define a true regulatory B cell subset.

In this paper, we show that induced IL-10 expressing B cells often co-express pro-inflammatory cytokines, which argues against a fully anti-inflammatory capacity of these cells. Although part of the cells produce only IL-10, these cells do not represent a single population as shown by variation of IL-10 expression level and do not express a unique surface marker signature. Finally, induced IL-10 expression is not stable: IL-10 producing B cells lose their capacity to produce IL-10 after re-stimulation. Taken together, these data suggest that *in vitro*-induced IL-10^+^ B cells do not represent a dedicated Breg subset but rather represents an activated B cell phenotype.

CpG stimulation resulted in the development of several populations of IL-10 producing cells, some of which display a distinctly pro-inflammatory phenotype, co-expressing TNFα and/or IL-6. Roughly half of the IL-10^+^ B cells express TNFα, while IL-6 expression was present in approximately 60% of IL-10^+^ B cells. Furthermore, 27–37% of IL-10 producing B cells co-expressed both cytokines. The population that expressed only IL-10 comprised approximately 25% of all IL-10 producing B cells. Co-expression of TNFα by IL-10^+^ B cells has been demonstrated before (37), but, since TNFα can also be associated with tolerance (43), this trait does not argue against an overall regulatory phenotype of these B cells. Our finding, however, of co-expression of the pro-inflammatory cytokine IL-6 does show that these cells also have pro-inflammatory properties, which may compromise the regulatory function of B cell-produced IL-10 on other cell types. Further research is needed.

Several subsets of B cells have been described to possess regulatory capacity by means of IL-10 production, especially transitional B cells (11) and plasmablasts (15). Within the transitional B cell population, the amount of IL-10^+^TNFα^−^IL-6^−^ B cells is enriched, with approximately 65% of IL-10^+^ B cells not producing TNFα or IL-6. However, IL-10 producing transitional B cells only constitute 0.19–0.36% of the CD19^+^ cells *in vitro*, whereas around 2% IL-10^+^ B cells are still present after IL-10^+^ B cells were sorted and re-cultured for 3 more days (Figure 6). This indicates that even though IL-10^+^ transitional B cells may be Bregs they are not the only possible Breg subset. In contrast, within the plasmablast pool, TNFα was the predominant cytokine, with only very few B cells expressing IL-10.

Since cytokine analysis of CpG stimulated cells indicates several distinct populations of IL-10^+^ B cells, unbiased clustering based on IL-10, TNFα, and IL-6 was performed using t-SNE analysis. This demonstrated not only that there are several populations of IL-10 producing cells based on cytokine production, but furthermore, that IL-10^+^TNFα^−^IL-6^−^ B cells are further divided into several distinct populations. However, no clear differences in surface marker expression were observed between these IL-10^+^TNFα^−^IL-6^−^ B cell populations.

Arguably, cells that produce IL-10 alone and not TNFα and/or IL-6 are more likely to have a regulatory function similar to dedicated Bregs. We therefore compared IL-10^+^TNFα^−^IL-6^−^ B cells to IL-10^+^TNFα^+^IL-6^+^ B cells using flow cytometry. Indeed, IL-10^+^TNFα^+^IL-6^+^ B cells display increased expression of CD80, indicating they might be more activated (44) and suggesting that IL-10 produced by these cells is a trait of activation. Interestingly, expression of CD5 was increased on IL-10^+^TNFα^−^IL-6^−^ B cells. CD5^+^ B cells in mice negatively regulate inflammatory responses (10). Although expression of CD5 was increased on IL-10 single-positive cells, compared to TNFα single-positive cells as well, expression of these markers was not significantly higher than on IL-6 single-positive cells arguing against this human CD5^+^ B cell subset possessing regulatory functions to dampen inflammatory responses. Furthermore, t-SNE clustering on all surface markers including IL-10, TNFα and IL-6 within IL-10^+^ B cells revealed a large overlap of IL-10^+^TNFα^−^IL-6^−^ with IL-10^+^ B cells co-expressing the pro-inflammatory cytokine IL-6 (IL-10^+^TNFα^−^IL-6^+^), indicating that within the markers we tested, all of which have been linked to regulatory B cells in literature, none of the markers, either alone or combined, can define a unique subset of IL-10 expressing B cells.

Dedicated Bregs should at least retain the capacity to produce IL-10 when (re-)stimulated. Murine B10 cells however, were shown to secrete IL-10 transiently (45). Therefore, IL-10 expression upon restimulation was investigated in our assay. Cells that produce IL-10 after induction with CpG lose IL-10 expression after 3 days of *in vitro* culture. When restimulated, only a small fraction of these cells produced IL-10 again. This fraction was comparable to the fraction of formerly IL-10^−^ cells that produced IL-10 after a second round of stimulation. Together with the co-expression of pro-inflammatory cytokines and the lack of defining Breg markers, this suggests that IL-10 production is a transient trait of activated B cells at specific time points during differentiation and may not be a true Breg subset identifier. To unequivocally assess regulatory capacity of the IL-10 B cells, suppression assays using the various IL-10-expressing B cells would be required. Currently, this is however, prohibited by the lack of surface markers distinguishing the IL-10^+^TNFα^−^IL-6^−^ B cells from B cells co-expressing TNFα and/or IL-6.

The finding that transitional B cells are relatively enriched within the IL-10-expressing population [Figure 2C and (11)] indicates that IL-10 expression is an early trait during B cell differentiation. Since IL-10 plays an active role in B cell differentiation, stimulating antibody production, expression of this cytokine after B cell stimulation is not surprising. The balance between dynamic production and consumption of IL-10 and the pro-inflammatory cytokines co-expressed by activated B cells, rather than the absolute amount of cytokine production, is likely to influence the outcome of local immunity. In this respect, it is of interest to mention the possibility for an autocrine loop, with IL-10 either suppressing or stimulating B cell activity (13); and may represent a general state of activation of the B cell.

The realization that IL-10-induction in B cells *in vitro* might not define a regulatory B cell subset *per se* but is part of an activated B cell phenotype co-expressing pro-inflammatory cytokines is important for the field of immunomonitoring and immunotherapy. Enrichment of IL-10 producing B cells *in vitro* in certain patient populations does not imply in itself that these patients exhibit overall immune regulation. Functional analysis of the regulatory capacities of IL-10^+^ B cells on the immunological effector function of various immune cells and the insight in stability of the IL-10^+^ B cells is warranted to get a better insight in the meaning of potential accumulation of IL-10 producing B cells in disease. The consideration of using induced IL-10^+^ B cells for tolerance-inducing therapies, either by itself or in combination with other tolerogenic cell types depends on the outcomes of these studies.

## Author contributions

LL and P-PU participated in acquisition of data, analyses and interpretation of data, drafting and writing of the article and approved the final version. DWV, DV, EV, and AT participated in acquisition of data, critically reviewed the article and approved the final version. AtB, TR, and SvH participated in design of research, interpretation of data and contributed to writing, reviewing and finalizing the article.

### Conflict of interest statement

The authors declare that the research was conducted in the absence of any commercial or financial relationships that could be construed as a potential conflict of interest.

## Abbreviations

Breg: regulatory B cell
hCG: human chorionic gonadotropin
BCR: B cell receptor
GCs: germinal centers
PBMCs: Peripheral blood mononucleated cells
FCS: fetal calf serum
PMA: phorbol 12-myristate 13-acetate
BFA: Brefeldin A
BSA: bovine serum albumin
t-SNE: t-distributed stochastic neighborhood embedding
DAPI, 4′: 6-diamidino-2-phenylindole.
