# Supplementary material for: In vitro-Induced Human IL-10+ B Cells Do Not Show a Subset-Defining Marker Signature and Plastically Co-express IL-10 With Pro-Inflammatory Cytokines
Source: Front Immunol. 2018 Sep 5;9:1913. doi: 10.3389/fimmu.2018.01913 (PMC6143818; doi:10.3389/fimmu.2018.01913)
Supplement: Supplementary file 1 [file Table_1.DOCX]

**Supplemental table 1**

List of used antibodies:

| **Antigenic target** | **Fluorochrome** | **Clone** | **Manufacturer** |
| --- | --- | --- | --- |
| CD24 | FITC | ML5 | BD Bioscience |
| CD38 | PE | HB7 | BD Bioscience |
| CD19 | BUV496 | SJ25C1 | BD Bioscience |
| CD80 | PECy7 | L307.4 | BD Bioscience |
| CD86 | BV421 | FUN-1 | BD Bioscience |
| CD27 | BV510 | O323 | BioLegend |
| CD25 | BV605 | 2A3 | BD Bioscience |
| CD40 | BUV395 | 5C3 | BD Bioscience |
| CXCR5 | PECy7 | MU5UBEE | eBioscience |
| CD48 | BV421 | TU145 | BD Bioscience |
| CD1d | BV510 | CD1d42 | BD Bioscience |
| CD5 | BV605 | UCHT2 | BD Bioscience |
| CD21 | BUV395 | B-ly4 | BD Bioscience |
| IL-10 | APC | JES3-19F1 | BD Bioscience |
| TNFα | PerCPCy5.5 | Mab11 | eBioscience |
| IL-6 | AF700 | MQ2-13A5 | eBioscience |

Antibody mixtures:

| **Mixture 1** | **Mixture 2** |
| --- | --- |
| IL-10 | IL-10 |
| TNFα | TNFα |
| IL-6 | IL-6 |
| CD24 | CD24 |
| CD38 | CD38 |
| CD19 | CD19 |
| CD80 | CXCR5 |
| CD86 | CD48 |
| CD27 | CD1d |
| CD25 | CD5 |
| CD40 | CD21 |
