# Supplementary figures and images for: In vitro-Induced Human IL-10+ B Cells Do Not Show a Subset-Defining Marker Signature and Plastically Co-express IL-10 With Pro-Inflammatory Cytokines
Source: Front Immunol. 2018 Sep 5;9:1913. doi: 10.3389/fimmu.2018.01913 (PMC6143818; doi:10.3389/fimmu.2018.01913)

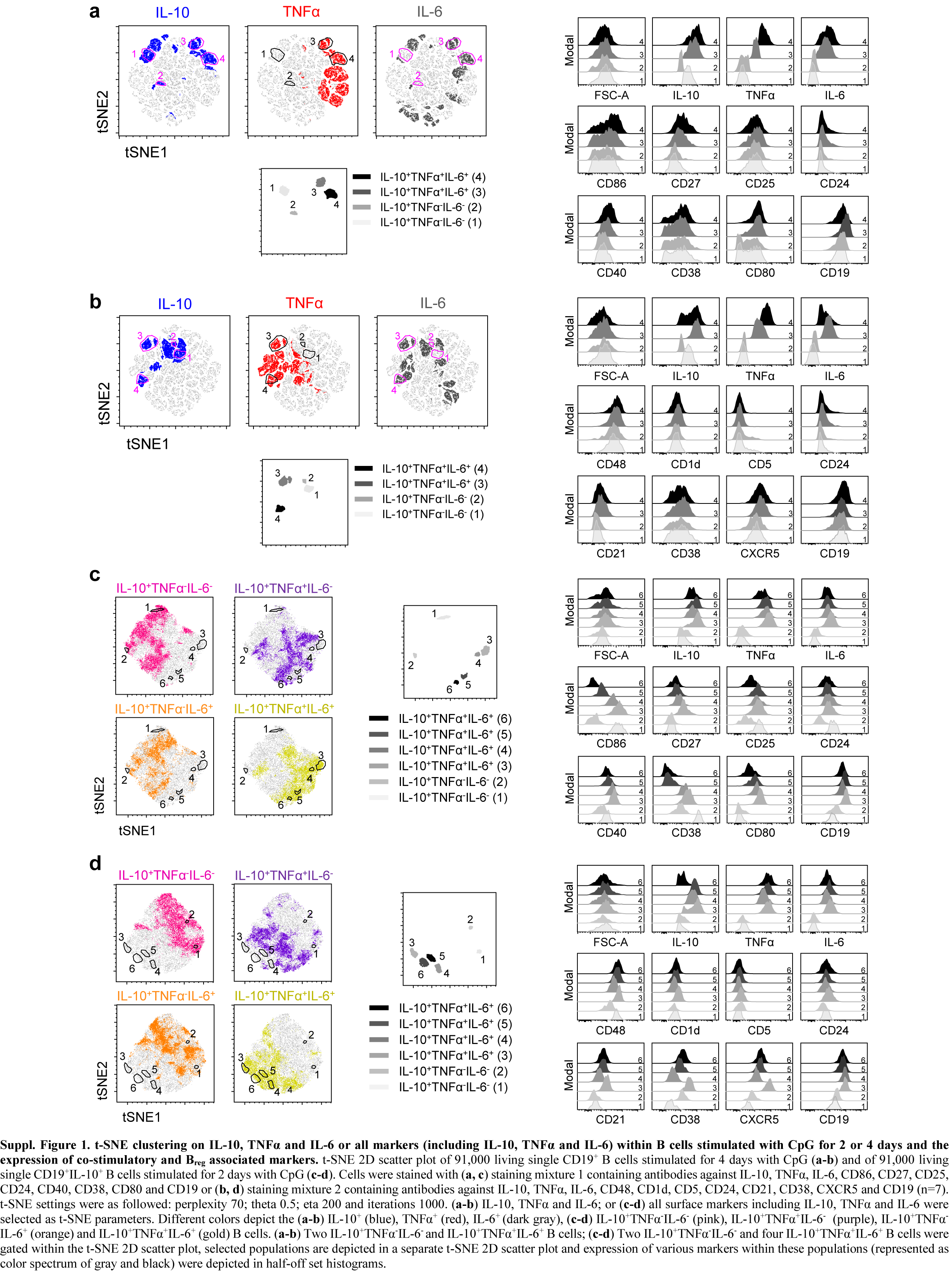

Supplement: Supplementary file 2 [file Image_1.tif]

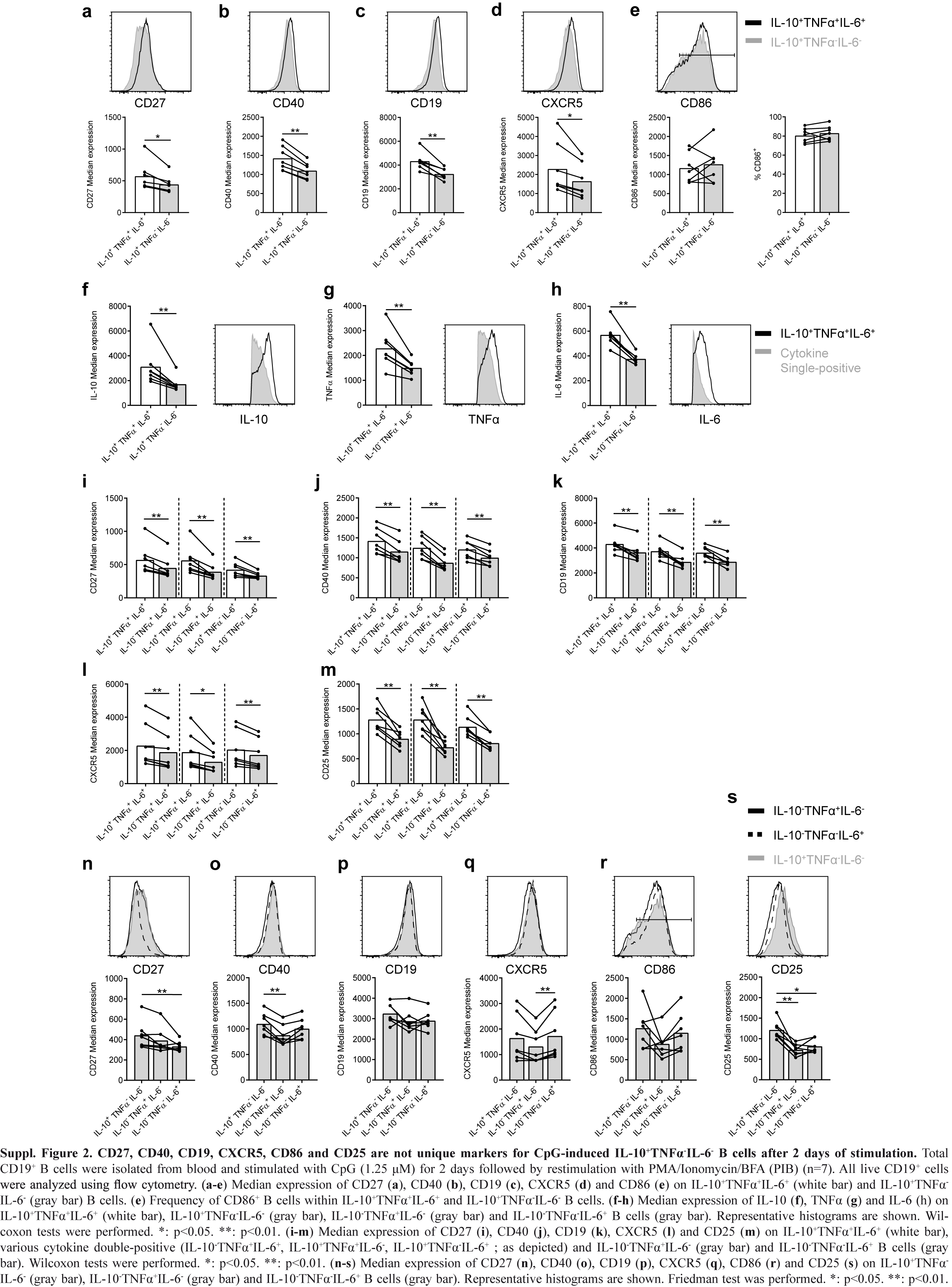

Supplement: Supplementary file 3 [file Image_2.tif]

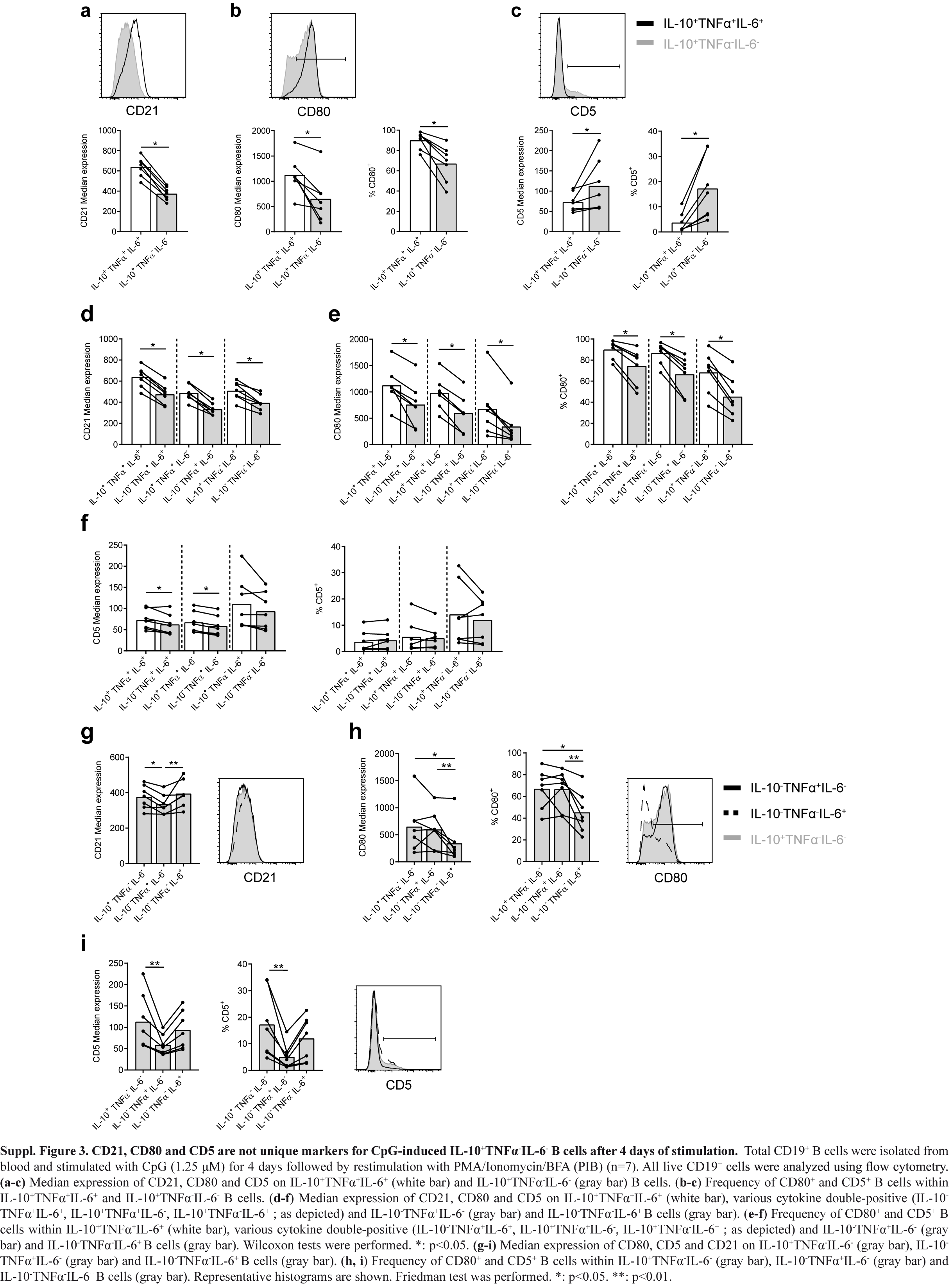

Supplement: Supplementary file 4 [file Image_3.tif]

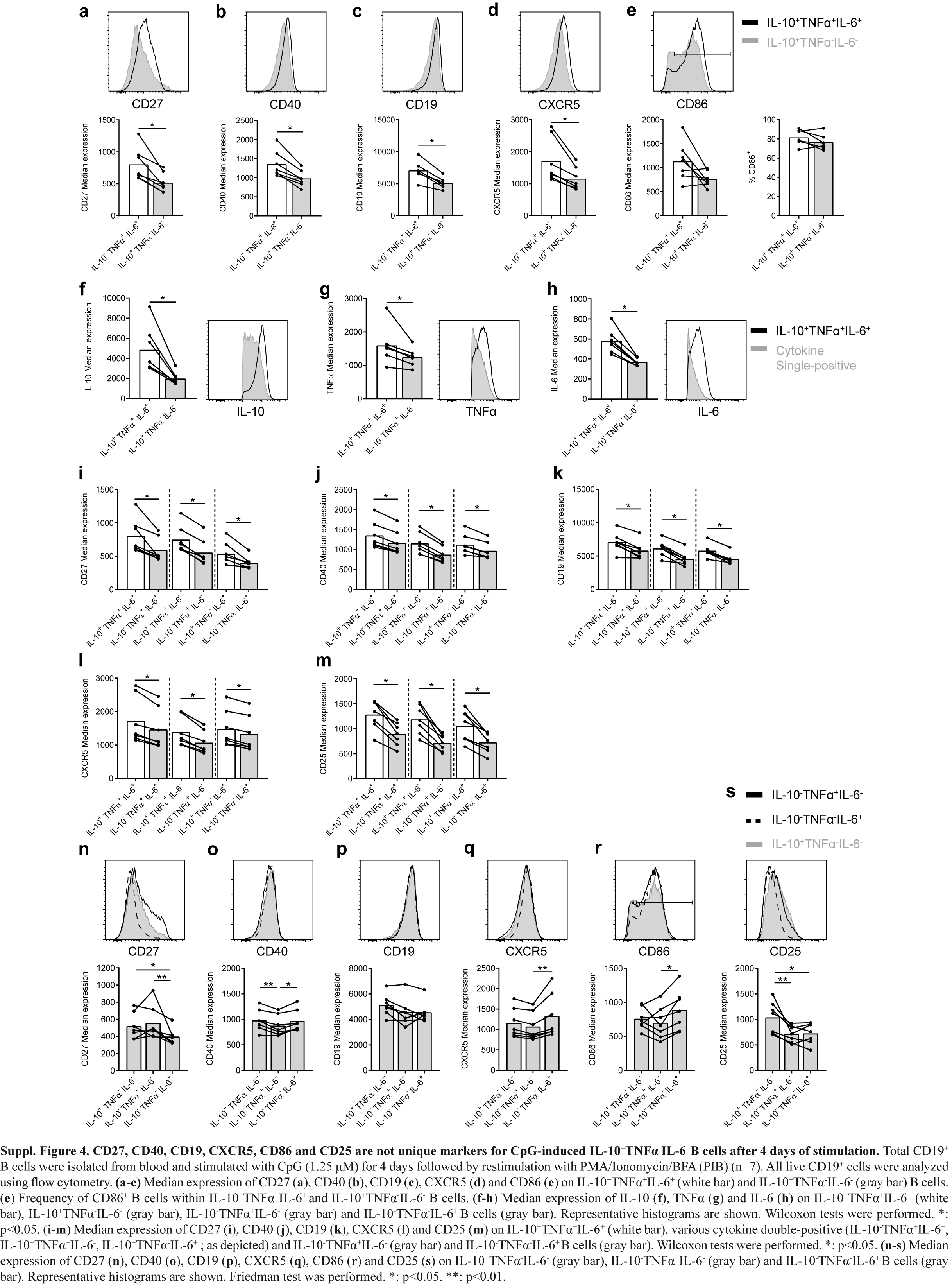

Supplement: Supplementary file 5 [file Image_4.tif]
